# DIRECT AND INDIRECT CAUSAL EFFECTS OF AIR POLLUTANTS ON ALZHEIMER’S DISEASE RISK AND DISPARITIES

**DOI:** 10.1093/geroni/igad104.2747

**Published:** 2023-12-21

**Authors:** Igor Akushevich, Arseniy Yashkin, Vito Di Bona, Julia Kravchenko

**Affiliations:** Duke University, Durham, North Carolina, United States; Duke University, Durham, North Carolina, United States; Duke University, Durham, North Carolina, United States; Duke University School of Medicine, Durham, North Carolina, United States

## Abstract

Ambient air contaminants have been associated with higher risk of Alzheimer’s disease (AD), related dementia, and cognitive impairment; however, mechanisms that underlay these effects remain unclear. Pre-existing arterial hypertension, diabetes mellitus, cerebrovascular disease, and renal disease have a strong impact on AD risk and disparities. These diseases, in turn, are associated with exposure to air contaminants, including particulate matter (PM2.5). We applied causal mediation analysis in the formulation of VanderWeele which provides causal estimates of the direct and indirect (through the effects on pre-existing diseases) effects of PM2.5 exposure on the risk of AD. We used Administrative Health Insurance Claims from 5%-Medicare (N=6,042,239; 1991-2020) to identify the date of onset for AD and risk related diseases such as arterial hypertension, diabetes, cerebrovascular, and kidney diseases that were considered mediators in these analyses. Information on ambient PM2.5 at the zip code level were derived from the Socioeconomic Data and Applications Center (SEDAC) that incorporates air monitoring data, satellite aerosol optical depth, meteorological conditions, chemical transport model simulations, and land-use variables. The estimates supported the hypotheses on the linear dependence of the dose-response function with higher effects for Males vs. Females, White vs. Black subpopulations, and stroke-belt states vs. other U.S. regions. We found that the contributions of indirect effects were stronger for younger older adults and reached up to 13% for cerebrovascular disease, 10% for arterial hypertension, 5% for diabetes, and 4% for chronic kidney disease. Analysis in subpopulations demonstrated health disparities in the effects of PM2.5 on AD risk.

